# Facial Affect Recognition and Executive Function Abnormalities in ADHD Subjects: An ERP Study

**DOI:** 10.1177/15500594241304492

**Published:** 2024-12-19

**Authors:** Saghar Vosough, Gian Candrian, Johannes Kasper, Hossam Abdel Rehim, Dominique Eich, Andreas Müller, Lutz Jäncke

**Affiliations:** 1 Division Neuropsychology, Department of Psychology, 27217 University of Zurich, Zurich, Switzerland; 2 399140 Brain and trauma foundation Grisons/Switzerland, Chur, Switzerland; 3 Praxisgemeinschaft für Psychiatrie und Psychotherapie, Lucerne, Switzerland; 4 Psychiatrie und Psychotherapie Rapperswil, Rapperswil, Switzerland; 5 Department of Psychiatry, Psychotherapy, and Psychosomatics, 27217 University of Zurich, Zurich, Switzerland

**Keywords:** attention-deficit hyperactivity disorder, VCPT, ECPT, ERP, facial affect recognition‌

## Abstract

Attention-deficit/hyperactivity disorder (ADHD) affects approximately 12% of children worldwide. With a 50% chance of persistence into adulthood and associations with impairments in various domains, including social and emotional ones, early diagnosis is crucial. The exact neural substrates of ADHD are still unclear. This study aimed to reassess the behavioral and neural metrics of executive functions and neural substrates of facial affect recognition. A total of 117 ADHD patients and 183 healthy controls were evaluated by two Go/NoGo tasks: the classic visual continuous performance test and the emotional continuous performance test, which requires facial affect encoding. Group differences between ADHD subjects and healthy controls were assessed using analysis of covariance (ANCOVA), with age and sex included as covariates. Dependent variables comprised behavioral (number of omission and commission errors, reaction time, and reaction time variability) and neurophysiological measures (event-related potentials [ERPs]). As the main result, we identified significant differences between ADHD patients and healthy controls in all behavioral metrics, one neural marker of action inhibition (P3d) and the facial processing marker (N170). The differences were moderate-to-large when expressed as effect size measures in behavioral variables and small-to-moderate for neurophysiological variables. The small-to-moderate effect sizes obtained from the neurophysiological measures suggest that ERPs are insufficient as sole markers for effectively screening emotion and face processing abnormalities in ADHD.

## Introduction

Attention-deficit/hyperactivity disorder (ADHD) is widely recognized as a neurodevelopmental disorder characterized by impairments in impulse control, hyperactivity, and attention deficits.^1,2^ ADHD typically begins before the age of seven (according to the ICD-10) or twelve (according to DSM-5)^3,4^ and affects approximately 8%-12% of children worldwide, with about a 50% chance of persisting into adulthood.^5–8^ Recent research highlights that ADHD is associated with difficulties in various social domains, such as social skills,^9^ interpreting social cues, maintaining eye contact, and developing peer relationships.^10–15^

Social cognition encompasses interconnected cognitive abilities required to process social information and navigate social interactions effectively. These abilities include affect recognition, pragmatic language, theory of mind (ToM), and empathy.^16^ Marsh and Williams specifically note that facial emotion encoding is particularly impaired in adults with ADHD.^17^ Dan further observes that individuals with ADHD require more time and make more errors than healthy controls when identifying facial emotions.^18^ Facial affect expressions should be encoded early in facial recognition to facilitate effective non-verbal communication, as they often convey biologically significant information.^19^ For example, an angry facial expression can heighten sensitivity to potential environmental dangers.^20,21^ Despite extensive research on the neurobiological substrates of ADHD,^22–26^ the underlying mechanisms of its symptoms remain unclear, making it one of the most debated diagnoses,^25,27^ and research specifically focusing on social cognition deficits in ADHD is even more limited and controversial.^16,28^

Many studies have examined the neural basis of ADHD using electroencephalogram (EEG), structural and functional MRI techniques.^29–35^ Several studies suggest a link between emotion processing and social cognition impairments, frontostriatal deficits, and executive functions (EF) in ADHD.^16,29,31,32,36,37^ In a study by Ibanez et al on adult ADHD, the N170 Event-Related Potential (ERP) component was used as a neurophysiological marker for facial emotion processing. The researchers also assessed EF and ToM. They found reduced N170 amplitudes in the ADHD group were associated with lower performance on an emotional inference ToM task and poorer EF.^38^

Several behavioral studies using standard Go/NoGo tasks have highlighted the involvement of neural structures in executive function (EF) control, revealing moderate-to-strong differences between ADHD subjects and healthy controls.^39–44^ ERP variants of the Go/NoGo paradigm are widely used to delineate the underlying neurophysiological processes.^43,45,46^ In the cueing variant of this paradigm, a Go-stimulus is preceded by a cue, allowing participants to prepare for a response. Occasionally, a NoGo-stimulus follows the cue, requiring them to withhold their response.^42,46,47^

Comparing the ERP responses to Go and NoGo stimuli reveals two dominant components: (a) the fronto-central N2d, associated with conflict monitoring, and (b) the fronto-central P3d, linked to action inhibition.^48–51^ Intracortical source modeling indicates that conflict detection is managed by the frontostriatal network, including the anterior cingulate and basal ganglia, while action inhibition involves the supplementary and pre-supplementary motor areas.^52–54^ As conflict detection and action inhibition are critical EFs affected in ADHD, ERP Go/NoGo tasks are frequently used to differentiate ADHD subjects from healthy controls. The N170 ERP component, generated from the fusiform gyrus and superior temporal sulcus, is a biomarker specifically linked to facial processing in many studies with normative populations and individuals with ADHD.^55,56^ This makes the N170 component an ideal marker for evaluating potential facial affect impairments in ADHD subjects.

This study re-assessed the prospect and extent of differences between ADHD subjects and healthy controls in standard behavioral and neurophysiological measures of EFs by two similar Go/NoGo tasks (visual continuous performance test [VCPT] and emotional continuous performance test [ECPT]). Based on existing literature,^46,57–59^ we hypothesized that ADHD subjects would demonstrate increased errors, reaction time (RT), ERP latencies, and attenuated ERP amplitudes. Several papers suggest a link between social cognition impairments and EFs in ADHD.^16,29,31,32,36,37^ Thus, we were particularly keen on the EF-related responses to the rarely used ECPT task,^60,61^ which, unlike VCPT, requires facial emotion recognition. We anticipated distinct performance in EF-associated measures of the ECPT task between the ADHD and control groups. Also, the study examined whether the face-sensitive N170 component differs between ADHD and control groups in the ECPT task, Go/NoGo conditions, and left/right hemispheres.

To our knowledge, no study has compared the neurophysiological and behavioral responses to these different Go/NoGo task variants in ADHD and healthy subjects.

## Methods

### Subjects

Data used in this research was obtained from a multicenter clinical and observational study by the Brain and Trauma Foundation Grisons, Switzerland. The data collection method has been extensively described in a study by Münger et al^62^ The Brain and Trauma Foundation project, with more than 674 participants, was approved by the cantonal ethics committee of Zurich (LeitEKZH_2013-0327/EKNZ_2014_160). In this project, certified psychiatrists and clinical psychologists diagnosed ADHD subjects according to the DSM-5. Several psychometric tests were administered.

This paper assessed the psychometric intelligence test using standard pen-and-pencil tests in the German language (≤ 9 years, CFT 1-R; 9 to 16 years, CFT 20-R part I^63^; > 16 years, WMT-2).^64^ The exclusion criteria for the study were traumatic brain injury, loss of consciousness in the past, primary mental disorders other than ADHD, drug abuse, pregnancy, epilepsy, and an IQ score below 80. The participants needed to be able to follow the study instructions in German and provide informed consent. All subjects were medication-free on the assessment day. However, 139 ADHD subjects (31%) had been taking methylphenidate-comprising medication daily (eg, Ritalin, Concerta, Elvanse). One hundred ninety-two subjects (43%) had not used methylphenidate. Medication information was missing for 116 of the subjects (26%).

We selected subjects with complete demographic information from this large sample who completed both ECPT and VCPT tasks. A total of 300 subjects (183 controls, 117 ADHD), aged 6-60 (M = 26.14, SD = 17), were analyzed. The sample comprised 89 children (age range 6-12 years; ADHD n = 57, controls n = 32), 43 adolescents (age range 13-18 years; ADHD n = 18, controls n = 25), and 168 adults (age >18 years; ADHD n = 108, controls n = 60). All subjects were drawn from the same pool as the Münger et al study, which reported only VCPT task results.^62^ This study compared the results obtained from both VCPT and ECPT tasks (Table 1).

**Table 1.** Descriptive Information of the Participating Subjects (n = 300).

	**Controls**	**ADHD**
N total	183	117
N (%), male	72 (39%)	74 (63%)
N (%), female	111 (60%)	43 (36%)
Age (mean ± SD)	29.23 (20.19)	24.22 (14.38)
IQ	105 ± 14	99 ± 16

Shown are the number of male and female subjects. In addition, the mean age and IQ (and standard deviation) for both groups are shown.

### EEG Recording and Processing

EEG data were recorded during the resting state (not reported here) and during the two Go/NoGo tasks controlled by a computer. EEG was recorded with a 23-channel digital EEG system (NeuroAmp^®^ x23 amplifier) with direct current coupling and 24-bit resolution (BEE Medic GmbH, Switzerland). The input signals were bandpass filtered (0.5 and 50 Hz) with a sampling rate of 500 Hz decimated to 250 Hz. Before data processing, the montage was changed from linked earlobes to common-average reference. Based on the international 10-20 system, the electrodes were placed using a fitting electrode cap with 19 tin electrodes (Electro-cap International Inc., USA). The impedance for all electrodes was kept below five kΩ.

Raw EEGs recorded using ERPrec software (BEE Medic GmbH, Switzerland) were processed using MATLAB-based in-house software. Eye blinks and horizontal eye movements recorded on Fp1, Fp2, T3, and T4 were automatically detected using the independent component analysis (ICA) decomposition and removed from the EEGs by zeroing the activation of the respective components. To remove the remaining artifacts, the filtered EEG segments with extreme activity in the 0-3 and 20-50 Hz frequency bands and segments with amplitudes larger than100 µV (defined as a threshold of six standard deviations above the mean for each channel) were rejected. The artifact-free data were further analyzed with a custom-built EEGlab plug-in.

### Go/NoGo Tasks

**Figure 1.**
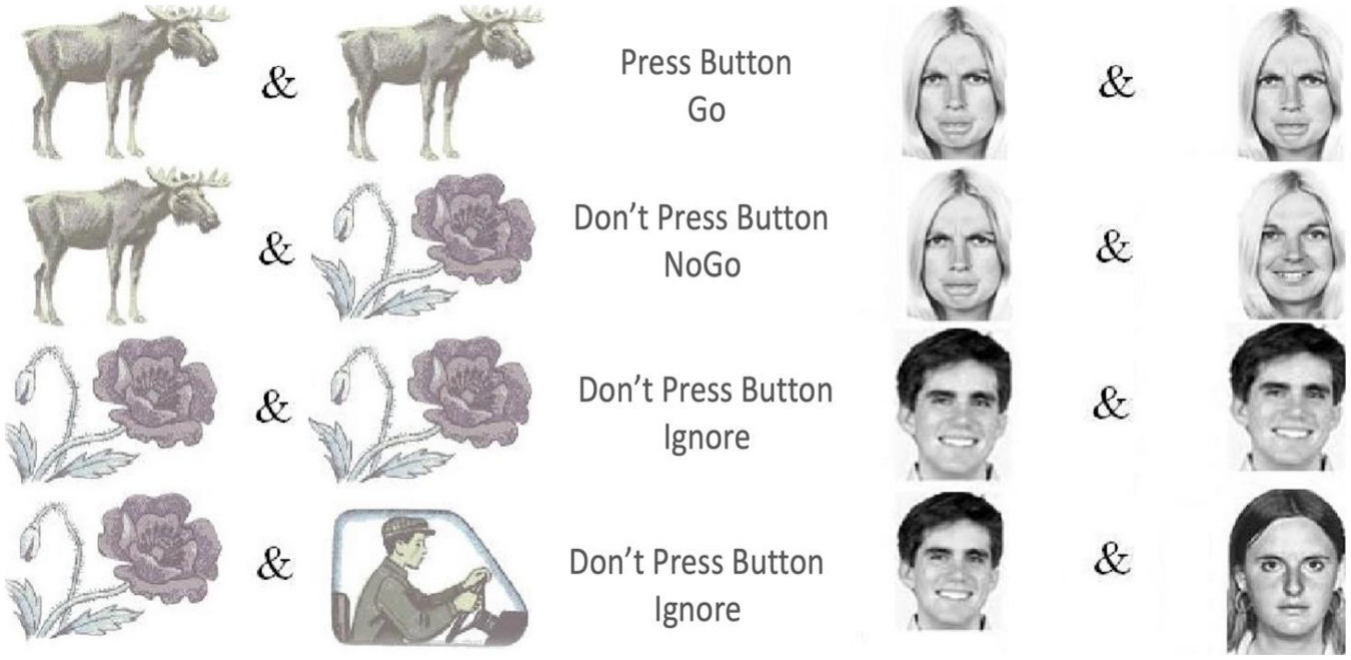
VCPT and ECPT conditions, including Go, NoGo, and distractor/ignore.

This study used two established Go/NoGo tasks: VCPT and ECPT.^60,61,65^ The VCPT task presents images of animals, plants, and humans as stimuli. A beep tone is presented simultaneously with the human images (Figure 1). In Go conditions, subjects were instructed to immediately click on the computer mouse when an image of an animal was followed by another image of an animal (animal-animal). In NoGo conditions, the subjects were instructed not to click on the mouse when an image of an animal was followed by an image of a plant (animal-plant). No action was required in the Ignore-conditions (images of plant-plant/human as stimuli).^66^

The ECPT task has a similar structure to the VCPT task, except for the content of the presented stimuli.^60,61^ In the ECPT task, the stimuli consist of images of female and male actors,^67^ with angry, happy, and neutral facial expressions. In Go-conditions, subjects were instructed to immediately click on the computer mouse when an image of an angry face was followed by another angry face image (angry-angry). In NoGo conditions, the subjects were instructed not to respond when an image of an angry face was followed by an image of a happy face (angry-happy). The Ignore-conditions required no action (images of happy-happy/neutral faces with a beep tone). Each condition consisted of 100 trials for 100 ms in both tasks, with an inter-trial interval of 3000 ms. The first stimulus was presented at 300 ms and the second at 1400. Each task lasted about 22 min (Figure 1).

In the behavioral measures, RT variability was calculated for Go conditions, defined as the coefficient of variance for RT. Omission errors (response failure in Go conditions) and commission errors (response failure in NoGo conditions) obtained from both CPT tasks were computed separately for each subject.

In the neurophysiological measures, three ERP components were assessed: the N2d as a marker of conflict detection, the P3d as the action inhibition marker,^49,68,69^ and the N170 as the face processing marker.^56,70–72^ The N2d and P3d are ERPs obtained during the Go trials minus the ERP obtained during the NoGo trials. The ERP latencies and amplitudes obtained during both tasks were set according to the maximum-peak method,^73^ using a MATLAB-based custom-built EEGLAB plug-in. After baseline correction using the 100 ms pre-stimulus period, the peak-detection method reliably detected the components of interest. The amplitudes were set at the highest peak on the ERP waveform in a time window whose size was adjusted to 80% of the time interval between our targeted component peak and the previous peak. For instance, if N2 is at 200 ms and P1 at 100 ms, the window for N2 was set from 160 to 240 ms. Self-modeling warping functions were applied to control the inter-individual temporal variability.^74^ All ERP components were measured where the waves are known to be most prominent. The N2d and P3d components were measured at the frontocentral (Cz), and the N170 was assessed in parieto-occipital scalp areas (T5-T6).^61,66,75–77^

### Statistical Analyses

For the behavioral measures, N2d and P3d ERP components, 2 *× *2 ANCOVAs were applied with *group* (ADHD, controls) as grouping factor and *task* (VCPT, ECPT) as repeated measurements factor. For the N170 component, a 2 *× *2 *× *2 ANCOVA was performed on the ECPT task with “*group”* (ADHD, controls) as the grouping factor, “*condition”* (Go, NoGo), and “*hemisphere”* (left, right) as repeated measurement factors. Dependent variables were the four behavioral measures (omission errors, commission errors, RT, and RT variability) and the six ERP amplitude and latency measures (N2d, P3d, and N170). We used sex and age as covariates in the ANCOVA analysis to control for their potential confounding effects and enhance our findings’ accuracy. By adjusting for these variables, we aimed to isolate the effects of the primary independent variables. The analysis results of sex and age variables are included in the ANCOVA results tables (Tables 2, 3, 4).

**Table 2.** Summary of the ANCOVA Results Separately for all Behavioral Variables Used as Dependent Variables. Age and Sex are Used as Covariates.

	Group		Task		Age		Sex		Group × Task		Age × Task		Sex × Task		
	*p*	η^2^G	*P*	η^2^G	*P*	η^2^G	*P*	η^2^G	*P*	η^2^G	*P*	η^2^G	*P*	η^2^G	*df*
VarRT	≤ .001	.15	≤ .001	.02	≤ .001	.03	.06	≤ .001	≤ .001	.001	.64	≤ .001	.21	≤ .001	294
RT	≤ .001	.05	≤ .001	.03	.06	≤ .001	.94	≤ .001	≤ .001	≤ .001	.18	≤ .001	≤ .001	≤ .001	294
Om	≤ .001	.11	≤ .001	.07	≤ .05	.01	.13	≤ .001	≤ .001	.02	≤ .02	≤ .001	≤ .001	≤ .001	294
Com	≤ .001	.02	≤ .001	.03	≤ .05	.01	.91	≤ .001	≤ .001	.002	.90	≤ .001	≤ .001	≤ .001	294

Shown are the ANCOVA results for the main effects *group* (ADHD and controls), *task* (VCPT and ECPT), and the interaction between both main effects (*group × task*). Depicted are the p-values, the effect sizes as generalized eta square values (η^2^G), and degrees of freedom (df).

VarRT: variability of reaction time; RT: reaction time; Om: Omission errors; Com: commission errors.

**Table 3.** Summary of the ANCOVAs Separately for the N2d and P3d ERP Component Measures (Amplitudes And Latencies).

	N2d				P3d				
	μV		ms		μV		ms		*df*
	*P*	η^2^G	*P*	η^2^G	*P*	η^2^G	*P*	η^2^G	294
Group	. 45	≤ .001	≤ .001	.02	≤ .001	.03	≤ .001	.02	294
Task	≤ .001	.06	≤ .001	.06	≤ .05	≤ .01	≤ .05	≤ .01	294
Age	≤ .001	.03	.06	.006	≤ .04	.01	≤ .03	.01	294
Sex	.06	.001	.94	≤ .001	.13	.006	.91	≤ .001	294
Group × Task	.61	≤ .001	≤ .05	≤ .001	.07	≤ .01	.14	≤ .001	294
Age × Task	≤ .001	.01	≤ .04	.005	.80	≤ .001	.76	≤ .001	294
Sex × Task	.94	≤ .001	.40	≤ .001	.29	≤ .001	≤ .05	≤ .003	294

Shown are the ANCOVA results for the main effects group (ADHD and control) and task (VCPT and ECPT) and the interaction between both main effects (group × task). The Age and Sex effects are also presented. Depicted are the *P*-values, the effect sizes as generalized eta square values (η^2^G) and the degrees of freedom (df).

**Table 4.** Summary of the ANCOVAs Separately for N170 ERP Amplitudes (in μV) and Latencies (in ms).

	N170 μV		N170 ms		df
	*P*	(η^2^G)	*P*	(η^2^G)	
Group	≤ .001	.03	≤ .001	.01	294
Task	≤ .001	.05	≤ .001	.01	294
Age	≤ .001	.05	≤ .001	.03	294
Sex	≤ .05	≤ .001	≤ .001	≤ .001	294
Group × Task	≤ .001	≤ .001	≤ .001	.01	294
Age × Task	≤ .001	.01	≤ .001	.01	294
Sex × Task	.76	≤ .001	.24	≤ .001	294
Condition	≤ .003	.001	.07	≤ .001	294
Group × Condition	.13	≤ .001	.18	≤ .001	294
Group × Condition × Task	.35	≤ .001	.07	≤ .001	294
Age × Condition	.22	≤ .001	≤ .001	≤ .001	294
Sex × Condition	.12	≤ .001	.12	≤ .001	294
Hemisphere	.38	≤ .001	.34	≤ .001	294
Group × Hemisphere	≤ .05	≤ .001	.93	≤ .001	294
Group × Task × Hemisphere	.91	≤ .001	.34	≤ .001	294
Group × Condition × Hemisphere	.09	≤ .001	.93	≤ .001	294
Age × Hemisphere	≤ .01	≤ .002	≤ .01	≤ .001	294
Sex × Hemisphere	.30	≤ .001	.34	≤ .001	294

Shown are the ANCOVA results (corrected for age and sex) for the main effects. *Group* (ADHD and control), *task* (VCPT and ECPT), *condition* (Go and NoGo), *hemisphere* (left and right), and the interactions between the main effects. Sex and Age effects are also represented. Depicted are the *P*-values and the effect sizes as generalized eta square values (η^2^G) and the degrees of freedom (df).

All statistical analyses were conducted in R (version 3.3.2, http://www.R-project.org). The “afex-package” in R was used to calculate the ANCOVAs.^78^ Since ten ANCOVAs were conducted, multiple testing was controlled using the Bonferroni-Holm procedure. (starting with .05/10 = .005).^79^ Moreover, since *P*-values depend on sample size, effect sizes were calculated for more accuracy.^80^ Effect sizes in the context of the ANCOVAs were given using the generalized η^2^ recommended for a repeated-measures design.^80^ A generalized η^2^ of more than .02 is considered a “small effect,” more than .06 a “moderate effect,” and more than .14 is considered a “large effect size.”^80,81^ Cohen's effect size was calculated for subsequent post-hoc tests. A d = .2 is considered a small effect, a d = .5 medium, and d = .8 represents a large effect size.^82^

## Results

### Behavioral Measures

The results of the ANCOVAs for the behavioral measures are shown in Table 2. We found significant *group × task* interactions for RT (η^2^g = .001) and omission errors (η^2^g = .02) with a small effect size. We conducted subsequent post-hoc tests using the Bonferroni-Holm correction to control for multiple comparisons. We confirmed that the ADHD group exhibited longer RTs and more omission errors than healthy subjects in the ECPT task, relative to the VCPT, with medium-to-large effect sizes as indicated by Cohen's d. (RT: t (294), *P* ≤ .001, d = .5; omission error: t (294), *P* ≤ .001, d = .8).

Since no *group × task* interaction effects were found for RT variability and commission errors, we examined the main *group* and *task* effects separately. There were significant *group* effects for both variables with strong effect sizes for RT variability (η^2^g = .14) and small effects for commission errors (η^2^g = .02). The obtained interactions were further tested for multiple comparison corrections. The results confirmed higher RT variability and commission errors for ADHD subjects. The adjusted *P*-values were very small, and effect sizes ranged from small to large (RT variability t (294), *P* ≤ .001, d = 1.01; commission errors t (294), *P* ≤ .001, d = .4). *Task* interactions had significant RT variability and commission errors effects (*P* ≤ .001), with effect sizes ranging from small to medium. (RT: η^2^g = .03, commission error: η^2^g = .03). The post-hoc tests characterized these effects by increased RT variability and commission errors in the ECPT task compared to the VCPT task across all subjects, with large effect sizes (RT variability t (294), *P* ≤ .001, d = 1.5; commission errors t (294), *P* ≤ .001, d = 1.2).

### ERP Measures

The results for the ANCOVAs for the N2d and P3d components are shown in Table 3. In the *group × task* interaction, we discovered a significant difference for N2d latencies with negligible power of effects (η^2^g = ≤ .001). The subsequent post-hoc tests confirmed longer N2d-latencies in the ADHD group during the ECPT task, in comparison to controls and to ADHD during the VCPT task with small-to-medium effect size (t (294) = 3.59, *P* ≤ .001, d = .4).

**Figure 2.**
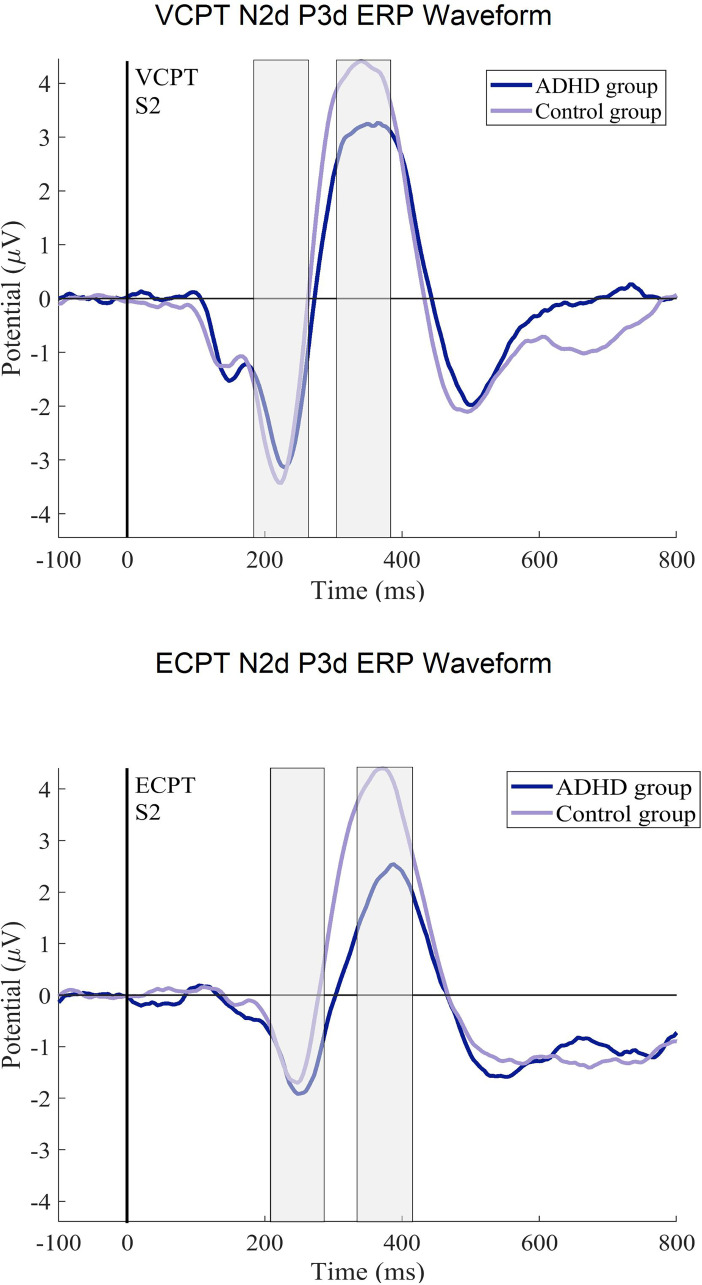
The ERP difference curves (N2d, P3d) for the ADHD and control groups. The ERPs after the second stimulus (at 1400 ms) in ECPT and VCPT are displayed.

**Figure 3.**
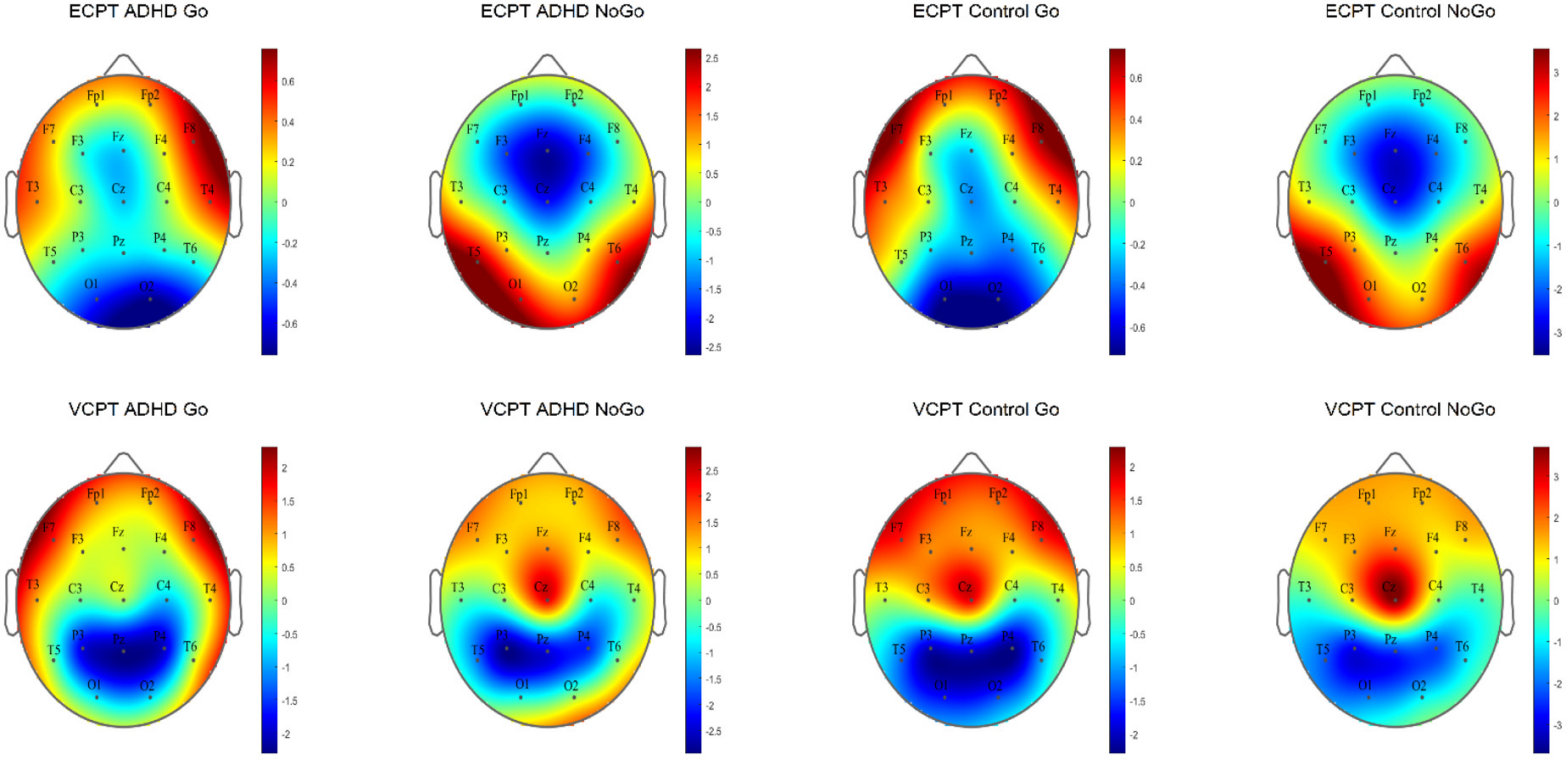
The N2d and P3d ERP topography plots for ADHD and control groups in ECPT and VCPT tasks are presented.

For the N2d-P3d amplitudes and P3d latencies variables with no significant *group × task* interactions, we checked for the *group* and *task* interactions separately. In *group* effects, we found significant differences in P3d amplitudes and latencies with a small power of effect (Table 3). In the subsequent post-hoc tests in P3d amplitudes, ADHD was confirmed to demonstrate lower amplitudes (t (294), *P* ≤ .001, d = .4) and longer latencies than healthy controls with small-to-medium effect sizes (t (294), *P* ≤ .001, d = .3). Figures 2 and 3 present the N2d and P3d ERP waveforms and topography plots of both groups in both tasks. In *task* interactions, we found significant effects in all N2d-P3d variables with small-to-medium effect sizes (Table 3), confirmed after multiple comparison corrections. In both N2d and P3d, all subjects demonstrated lower amplitudes in the ECPT task compared to the VCPT task with large and medium effect sizes (N2d t (294), *P* ≤ .001, d = 1.2; P3d t (294), *P* ≤ .001, d = .5). In P3d latencies all subjects presented longer latencies in the ECPT task than the VCPT with large magnitude of effects (t (294), *P* ≤ .001, d = .8).

**Figure 4.**
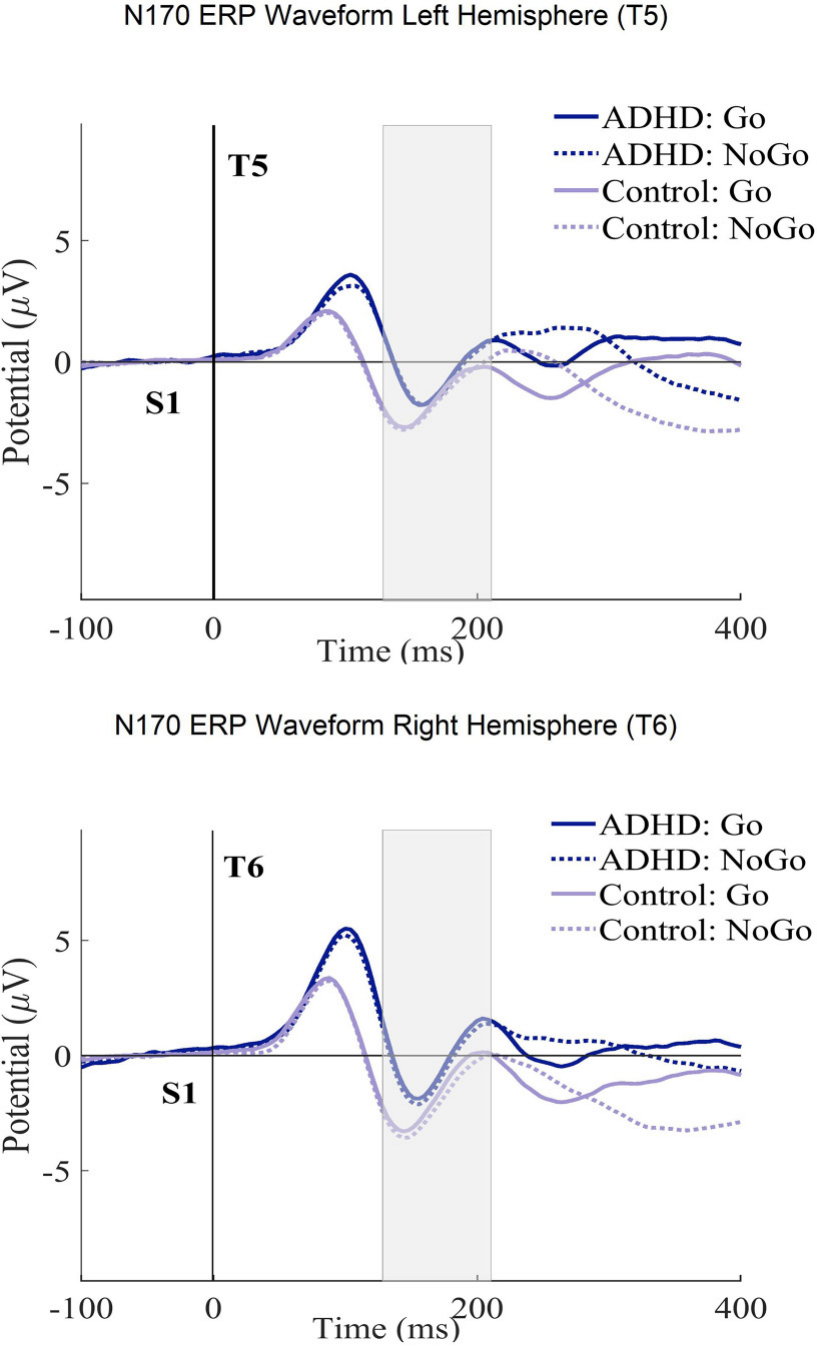
The N170 ERP curves in ECPT and VCPT after the second stimulus (at 1400 ms) for both T5-T6 (left-right hemispheres) electrode sites in groups (ADHD and control) and conditions (Go and NoGo) are presented.

**Figure 5.**
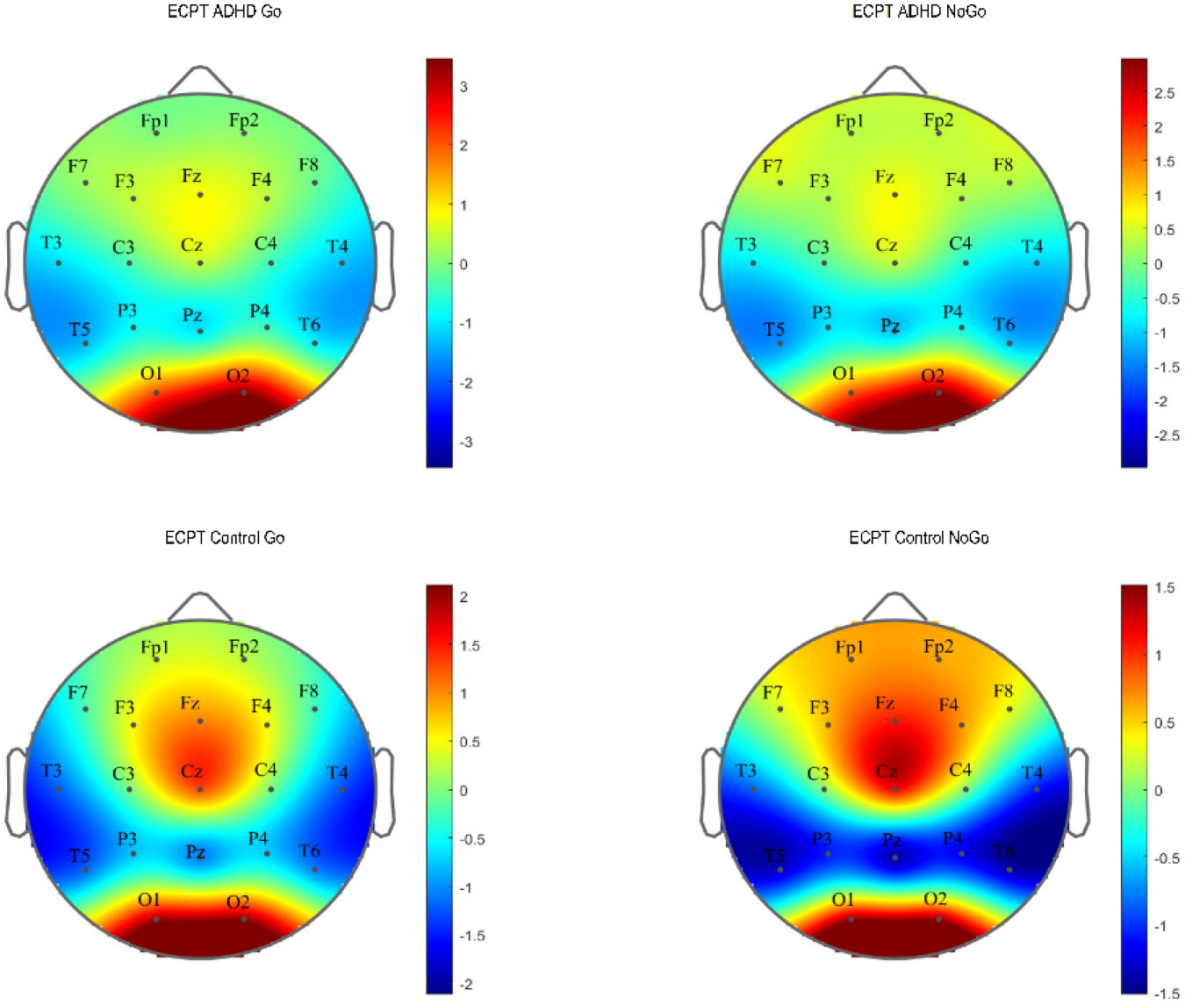
N170 ERP topography plot in the ECPT and VCPT tasks between groups (ADHD and control) and conditions (Go and NoGo).

Table 4 shows the ANCOVA results with the N170 amplitudes and latencies in the ECPT task. For the *group* interactions, only the N170 amplitudes met the significance threshold (*P* ≤ .04) with a small effect size (η^2^g ≤ .01). The consecutive post-hoc tests could successfully confirm these results qualified by the ADHD presenting significantly lower amplitudes than healthy controls with a small Cohen's magnitude of effects (t (294), *P* ≤ .01, d = .02). No significant differences were found between subjects for the *condition* and *hemisphere* interactions. For a better overview, the N170 ERP waveforms and topography plots are presented in both groups for each condition and hemisphere in Figures 4 and 5.

## Discussion

The present study combined behavioral and brain function measures to re-assess behavioral and neurophysiological EF parameter differences between ADHD patients and healthy controls. It also compared the findings of facial emotion processing in ADHD and healthy controls.

For behavioral measures, *group × task* interactions, which reflect the group differences across tasks, confirmed that ADHD subjects demonstrate significant deficiencies in RT and omission errors in the ECPT task (Table 2). Our findings were in line with several ADHD studies, indicating that ADHD individuals have impaired basic cognitive processing, as seen by their higher level of distractibility and inefficient processing speed.^83–89^ The RT is linked with the decision-making speed and reflects the interval between the stimulus presentation and the individual's response.^90^ Omission error is linked with lapses of attention and distractibility.^43,91,92^ RT and performance accuracy combined are suggested to represent poor basic information processing rather than separate impacts of executive dysfunctions in children with ADHD, as demonstrated by Metin et al^93^ and Salum et al^94^ The slow rate of information accumulation suggests that distinguishing signals from noise may be challenging, which could account for the delayed RTs and higher frequency of errors in ADHD performance. Ultimately, the RT lag and higher omission errors in ADHD compared to controls during the ECPT task may suggest that these processes are more impaired during the higher cognitive demand of face processing combined with EF and could contribute to a more in-depth understanding of the facial emotion processing impairments in ADHD. Additionally, in the *group* effects, ADHD showed higher reaction-time variability with a large effect size (η^2^g = .14) and higher commission errors with a small magnitude of effects (η^2^g = .2). Our findings align with the literature suggesting RT variability as one of the most reliable markers in ADHD diagnosis, not only distinguishing ADHD from healthy individuals but also from other psychiatric disorders.^42,95–98^ Commission error is associated with one of the core ADHD symptoms: response control and inhibition deficit.^47,85,89,92,99–104^ Identifying significant *group* interactions in RT variability and commission errors, as opposed to *group × task* interactions, suggests that these impairments are independent of face processing in individuals with ADHD.

In the ANCOVA analysis for the neurophysiological metrics of EF (N2d-P3d), only the N2d latencies were proven to be significantly different in the *group × task* interactions (Table 3). These differences were qualified by the ADHD showing longer latencies than controls in the ECPT task compared to VCPT. These results indicate that facial emotion processing is slower in ADHD. However, the small magnitude of effects suggests these impairments to be mild (η^2^g ≤ .001). The N2d reflects conflict detection and monitoring^23,105^ and is considered a neurophysiological marker of EF.^106–108^ EFs are essential in the higher-order mental processes that monitor human thoughts, emotions, and behavior.^109^ It is also suggested that the development of EF is closely linked to ToM development.^110–112^ These psychological functions share some common neural underpinnings, specifically involving prefrontal cortical regions.^113^ ToM is the ability to understand the beliefs, intentions, and emotions of others.^114–116^ The link between the EF and ToM might explain why ADHD subjects demonstrate more difficulties with tasks that require emotion encoding. Another explanation might be that the more diverse images presented as stimuli in the VCPT task (animals, humans, plants) compared to the ECPT task (facial expressions) can potentially cause more conflict monitoring in the brain, which nonetheless is an indicator of executive dysfunction in ADHD. In *group* effects, we found significantly lower amplitudes and longer latencies in P3d as a neurophysiological marker of response inhibition.^59,66,75,76,106,117–119^ The attenuated P3d amplitudes and longer latencies observed in individuals with ADHD across both tasks confirm previous findings on ADHD and executive dysfunction, suggesting that these impairments are independent of deficits in emotion recognition.^59,66,75,76,106,117–119^

In the facial-encoding-sensitive N170 component, the ADHD subjects demonstrated significantly lower amplitudes than healthy subjects (Table 4). This finding points to a particular deficit in facial affect processing and confirms the previous findings.^70–72,120–122^ The fusiform gyrus is considered one of the main origins of N170.^55^ Several studies assume that the fusiform gyrus is connected to the amygdala, a fundamental neural structure for processing negative emotions and social cognition.^123–125^ These two structures contribute to an emotion-processing network.^126–128^ Vuilleumier et al^129,130^ have demonstrated increased fusiform gyrus activation for emotional faces stemming from direct amygdala inputs. The amygdala of individuals with ADHD has appeared to have abnormalities.^22,123,127,128,131,132^ N170 does not directly measure amygdala activity. However, the reduced amplitudes in ADHD subjects during the ECPT task could be linked to amygdala–fusiform connection impairments, leading to social problems in ADHD. The small effect size suggests the mentioned impairments are mild. We found no significant *condition* and *hemisphere* interactions. Our findings differed from some studies on facial processing using the ECPT task.^15,72,133^ However, they were consistent with another similar study with a smaller sample size.^61^ These inconsistencies can be explained by differences in the task designs and electrode placements used to examine facial affect processing. Since the N170 is associated with sensitivity to the long-term familiarity of faces,^115–119^ we controlled for this effect by comparing the first and last 50 trials for each condition but found no significant effects (μV (*P* = .31), ms (*P* = .16)).

Our findings confirm the differences between ADHD subjects and healthy controls in behavioral markers of EF. This study found that individuals with ADHD show deficiencies in facial affect recognition. However, the differences obtained from the neuropsychological markers have small-to-moderate effect sizes, suggesting the processes behind these markers are mildly impaired in ADHD.

## Conclusion and Limitations

The data for this project were obtained in a clinical setting. We did our best to control the data acquisition as precisely as possible. However, clinical settings are often associated with greater variance during data acquisition than strictly controlled laboratory experiments. Another identified issue, also present in other studies, was that the diagnosis of ADHD is entirely based on subjective criteria with low reliability and validity.^134^ A misdiagnosis of nonverbal learning disorder and ADHD is sometimes made on this basis.^135–137^ Comparing ADHD and control subjects based on ERP components strongly suffers from this issue.

In addition, our clinical and control samples were heterogeneous regarding age and gender.

This study analyzed a relatively large clinical dataset with a wide age range. The adjusted group differences were assessed using two CPT tasks involving different neurophysiological processes. In behavioral metrics of EF, significant group differences were found with a moderate-to-large magnitude of effects (Table 2) and small-to-moderate effects in the N2d-latency component (Table 3). Our findings on the N170 component imply that ADHD subjects have deficiencies in facial emotion recognition (Table 4). Overall, the lower values demonstrated by the ADHD group in the N170 component and in measures associated with EF while processing facial emotions add evidence to the premise of a link between emotion processing and EF impairments in ADHD.^16,29,31,32,36,37^ However, the small-to-moderate effect sizes in neuropsychological measures suggest that ERPs are insufficient as exclusive markers for effective screening of emotion processing and EF deficits in ADHD without comprehensive data, such as behavioral measurements. This is especially true for clinical studies such as ours. In tightly controlled experimental settings, the findings may be different.

While our study did not differentiate the ADHD subtypes, some studies suggest that different subtypes (inattentive, hyperactive, and combined)^3^ have different EEG profiles.^132,133^ One major limitation is the wide age range (6-60 years) used to calculate the grand ERP averages in Figures 2 to 5. ERPs and their components are known to undergo substantial changes across this age span, especially between children and adults.^138–140^ These developmental changes in brain function can potentially introduce variability that may obscure or confound the effects observed in our study.^138,140,141^ Thus, the ERP figures should be considered carefully. Although analyzing a wide age range offers benefits, such as a broad overview of ERP patterns and identifying age-independent interactions, future research could focus on age-specific analyses or subdivide the age range. This would allow for a clearer understanding of ERP development in ADHD and healthy controls during face processing, potentially revealing age-specific patterns missed in the current analysis

Ultimately, previous attempts at finding neurophysiological markers of ADHD suggest a cautious approach to their application.^125–133^ However, this does not mean the search for easily measurable biomarkers is obsolete. Simple neurophysiological characteristics may need to be evaluated by new mathematical methods to detect even minor neurophysiological abnormalities. Initial approaches to detecting neurophysiological problems that may suggest the basis of psychological problems have already been presented.^142–144^ The application of these similar findings in ADHD research remains to be explored.
